# Enhancing Breast Cancer Detection Through Optimized Thermal Image Analysis Using PRMS-Net Deep Learning Approach

**DOI:** 10.1007/s10278-025-01465-y

**Published:** 2025-05-06

**Authors:** Mudassir Khan, Mazliham Mohd Su’ud, Muhammad Mansoor Alam, Shaik Karimullah, Fahimuddin Shaik, Fazli Subhan

**Affiliations:** 1 Department of Computer Science, College of Computer Science, Applied College Tanumah, King Khalid University, P.O. Box: 960, 61421 Abha, Saudi Arabia; 2 Faculty of Computing and Informatics, Multimedia University, 63100 Cyberjaya, Malaysia; 3 Faculty of Computing, Riphah International University, Islamabad, 44000 Pakistan; 4 Department of Electronics and Communications Engineering, Annamacharya University, Rajampet, Kadapa, Andhra Pradesh India 516126; 5 Faculty of Engineering and Computer Science, National University of Modern Languages, Islamabad, 44000 Pakistan

**Keywords:** Breast cancer, Early detection, Progressive residual networks, ResNet-50, PRMS-Net, Fivefold cross-validation, Medical imaging, Diagnostic accuracy, Machine learning, Feature extraction, Image classification

## Abstract

Breast cancer has remained one of the most frequent and life-threatening cancers in females globally, putting emphasis on better diagnostics in its early stages to solve the problem of therapy effectiveness and survival. This work enhances the assessment of breast cancer by employing progressive residual networks (PRN) and ResNet-50 within the framework of Progressive Residual Multi-Class Support Vector Machine-Net. Built on concepts of deep learning, this creative integration optimizes feature extraction and raises the bar for classification effectiveness, earning an almost perfect 99.63% on our tests. These findings indicate that PRMS-Net can serve as an efficient and reliable diagnostic tool for early breast cancer detection, aiding radiologists in improving diagnostic accuracy and reducing false positives. The separation of the data into different segments is possible to determine the architecture’s reliability using the fivefold cross-validation approach. The total variability of precision, recall, and F1 scores clearly depicted in the box plot also endorse the competency of the model for marking proper sensitivity and specificity—highly required for combating false positive and false negative cases in real clinical practice. The evaluation of error distribution strengthens the model’s rationale by giving validation of practical application in medical contexts of image processing. The high levels of feature extraction sensitivity together with highly sophisticated classification methods make PRMS-Net a powerful tool that can be used in improving the early detection of breast cancer and subsequent patient prognosis.

## Introduction

Mammary carcinoma is the most common type of cancer in the world and a major threat to women’s health [1–5]. It begins in the breast tissue and is often found in the milk ducts or the lobules which are the glands that nourish these ducts with milk [6]. Breast cancer health risks are numerous as it can lead to complications, and if not diagnosed at an early stage, lethal [7–12]. It is normally characterized by a rapid progression to other organs, hence the need to diagnose and start the treatment at an early stage to increase the disease’s survival rates [13].

It is important to mention, that the early diagnosis of breast cancer can increase treatment effectiveness and patient’s survival substantially [14–17]. Cancer of the breast is the commonest cause of cancer deaths in women; yearly, half a million women die from the disease globally, according to WHO [18–20]. Approximately 627,000 deaths occurred from breast cancer among women in 2018, of which breast cancer is 15% of all cancer deaths among women. Current detection strategies call for early and frequent screening and the use of diagnostic imaging techniques to identify the disease in its early stage [21]. Due to the early diagnosis when it has not reached the clinically detectable stage, the management and handling of the disease are much easier, more efficient, less aggressive to the body, and enhance the patient’s livelihood [22].

Medical imaging and machine learning progress to new methods, which provide better early diagnosis of breast cancer [23]. Thermal imaging is one of those promising technologies out there. While conventional breast cancer screening involves physical touch to feel for lumps, thermal imaging employs infrared technology to provide clues that there is cancer to the breasts’ temperature [24]. This method is somewhat advantageous since it does not involve a biopsy of the lesion, is not painful, and does not expose the patient to radiation. The differences in heat patterns are useful in determining whether the tissues are active, and if so, whether this activity is pathologic as cancer.

The novelty of the existing work based on the PRMS-Net model is that the progressive residual networks (PRN) have been incorporated together with the ResNet-50 redesigned to work with the thermal images for breast cancer detection. This model takes it a notch higher, by using deep learning to boost feature extraction and increase classification accuracy significantly.

## Related Works

 In a series of studies conducted in 2024, multiple methods were used to learn better about cancer or improve the technology involved in cancer detection by way of imaging and computation although each had its own inherent drawbacks depending on which model was used. The authors in [25] employed a modified CNN along with FCM for the classification of breast cancer in thermal images with 96.8% accuracy and specificity of 93.7%. The weakness of the model is that, because of intricate integration with Fuzzy C-means, it can easily over-fit the interactions and thus may not work well with different thermal imaging settings or different sets of patient populations.

Geetha et al. [26] developed a multiwavelet-based deep learning framework with an accuracy of 98 and precision and recall of 99% and 97.43% respectively. The major limitation of this method is that it is based on multiwavelet transformation which needs significant computer resources, and it might not be feasible for real-time systems where speed is very important.

K-NN and, in particular, an integrated KNN-SVM model allowed Moayedi et al. [27] to reach a level of accuracy of 98.8%; at the same time, sensitivity and specificity were equally high, both numbers being above 98%. The disadvantage is here due to the fact that the model’s performance depends on the quality and distribution of data. In the case of the asymmetrical distribution sampling patterns including data distributions, there are chances that the efficiency of the combined KNN + SVM decreases and returns biased or incorrect results of studies.

Yerken et al. [28] used explainable AI (XAI) as an approach to make the desired decision transparent and obtained 90.93% accuracy and 90.6% precision. It does so at a computational cost of the algorithm’s complexity and may not necessarily margin for high accuracy rates characteristic of “black boxed” models, making the use of XAI in clinical practice where interpretability does not compromise on high and accurate results less feasible.

Davies et al. [29] used MobileNetV2, with which they obtained an accuracy of 98.69 percent and a similar F1 score. The first weak point common to all MobileNetV2 is its application-oriented design, which may not process intricate or volatile data as efficiently as more resilient networks, which might portend its diminished functionality in a diverse clinical setting.

Rathi et al. [30] selected VGG16 because of its depth and complexity; due to these features, it provided an accuracy of 99%. But VGG16 needs vast computational power and often overfits, especially if experimented on low variance datasets could prove to be a limitation for applying the same in less controlled settings.

Years later, in 2023, Rezazadeh et al. [31] used CNN but with SVM and KNN together and reached even 95% accuracy, with sensitivity and specificity slightly higher. The enhancement of the model using multiple algorithms will add more layers to the complexity making it a difficult model to compute, and parameter tuning becomes an issue which becomes a hindrance in the practical application of the model.

Alia et al. proposed a deep learning-based approach to classify the type of breast lesion using convolutional neural networks (CNNs) that incorporated attention mechanisms to pay attention to the relevant features for the classification of types of breast lesion, and the study yielded an accuracy of 99.49% and F1-score of 99.36% [32]. Nevertheless, the specific attention mechanisms improve the focus and accuracy of the model and, at the same time, reduce its flexibility due to focusing on carefully selected and cleaned data inputs and increased demands toward available computational capacities, which might be limited in various healthcare environments.

Some of the typical limitations of using the advanced cancer detection methodologies as demonstrated in the reviewed studies above and summarized in Table 1 include overfitting, high computational complexity, inherent sensitivity to data quality and distribution, and practicality in real-time implementations. Here various constraints are often inherent in the intrinsic design of the models like high computation power requirement for any model like VGG16 and Multiwavelet-based deep learning; there will be bias in the outcomes that are sensitive to the unbalanced datasets and hence models such as the KNN+SVM combination.

**Table 1:** Summary of reviewed works with limitations

Authors	Year	Methodology	Specific limitations
Dharani et al	2024	Enhanced CNN with Fuzzy C-means	Potential for overfitting; may not generalize across setups
Geetha et al	2024	Multiwavelet-based deep learning	Requires significant computational resources; not practical for real-time applications
Moayedi et al	2024	KNN + SVM	Performance affected by unbalanced datasets
Yerken et al	2024	Explainable AI (XAI)	Computational intensity; lower accuracy than more opaque models
Davies et al	2024	MobileNetV2	Less effective in handling complex or variable data
Rathi et al	2024	VGG16	High computational demands; prone to overfitting
Rezazadeh et al	2023	CNN + SVM + KNN	Increased complexity and computational demand
Alia et al	2022	Deep learning with attention mechanisms	Dependent on high-quality data and computational power

To overcome these limitations, the present work proposes a new architecture named PRMS-Net which combines progressive residual networks (PRN) with ResNet-50. In the PRN part of PRMS-Net, feature extraction is improved due to sophisticated residual learning that makes the network study residual differences between inputs and outputs be predicted. This is especially helpful in preserving important feature information with the problem of network depth where the network’s depth causes the network to lose out in performance. Similarly, ResNet-50 employs a multi-layered feature extraction process that allows the model to extract simple as well as intricate features in the data set. This dual approach also tackles the problem of vanishing gradients because it allows for better gradient flow in the network while increasing the roughness and accuracy of the model. Oversampling of the receptor planes is also advantageous in assessing thermal images as thin differences are important in the imagery. The integration of PRN and ResNet-50 in PRMS-Net completely mitigates many of the mentioned drawbacks and provides a strong solution for more accurate and efficient detection of cancers.

## Proposed System

The PRMS-Net integrating the PRN and the ResNet-50 network enhances the potential of correctly detecting breast cancer through thermal imaging. This working methodology adopts features from both PRN and ResNet 50 to form a highly specialized clinical diagnostic tool for the evaluation of dense thermal data patterns with high precision. PRN is particularly perceived to improve deep learning models by allowing the learning of detailed patterns from data without being caught in the depth of the network. This network helps in gauging high-frequency features in thermal images that include early markers of breast cancer. Here PRN’s residual learning approach enhances PRN’s capacity to address problems in training deep networks, such as vanishing gradients to guarantee the network learns as it gains depth. The hierarchical feature extraction method embedded in ResNet-50 augments the operations of PRN. ResNet-50 has many convolution layers and can identify mutual low-level and high-level features through the thermal image, and it provides the much-enhanced data that enhances the classification process’s accuracy. Thereby, this synergy between PRN and ResNet-50 helps the model learn more composite features and brings a better level of accuracy in detecting abnormalities than ordinary imaging approaches.

### Proposed Methodology Diagram and Discussion

**Fig. 1:**
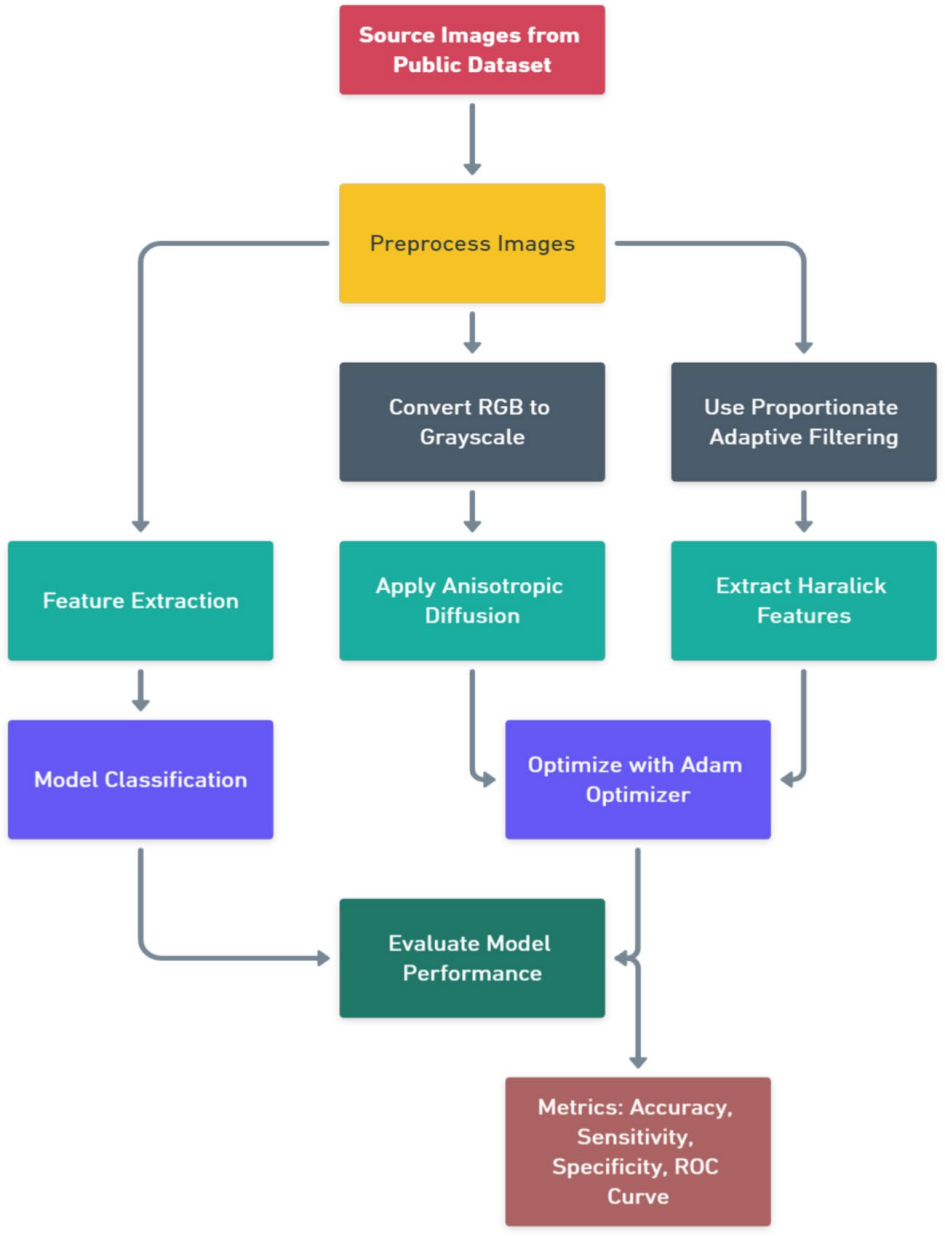
Proposed methodology block diagram

Figure 1 depicts an image classification pipeline, starting with data preprocessing, including grayscale conversion and adaptive filtering. Feature extraction involves anisotropic diffusion and Haralick features, followed by Adam optimization. The model is then classified and evaluated using accuracy, sensitivity, specificity, and ROC curves, ensuring robust performance and reliability.

For image processing, converting the image to grayscale from a color image (RGB) makes it easier for analyses but does not remove the structures. Normally, a thermal image does not have to have all the RGB channels for analysis since normally temperature differences have to be seen through differences in the intensity rather than the color. This transformation brings down the dimensions of the image from 3D (R, G, B) to 1D, that is the intensity of the image. As explained in the case of the weights, the coefficients correspond with human vision, and people are more susceptible to green hue; hence, there is a greater green channel weight. By converting the image into black and white color, the pictorial complexity is reduced and therefore helps visualize the structural properties of the image such as edges or the warm and cooler areas in the image.

Typically, thermal images expose contrast which in most cases reduces the chances of amplifying the differences in the image anomalies. Histogram equalization was found to be of immense help in making the pixel intensities spread out to reach the highest and lowest values possible leading to increased contrast. Double-exponential histogram equalization (DEHE) intensifies contrast modification by using a double-exponential function which enhances both low and high contrast regions.

Given an image’s pixel intensities I(*x*, *y*), the histogram equalization function is computed as shown in Eqs. (1) and (2):

1 $${p}_{i}= \frac{{n}_{i}}{N}$$

2 $$cdf(i)=\sum_{j=0}^{i}{p}_{j}$$

where ${n}_{i}$ is the number of pixels with intensity, *N* is the total number of pixels, and $cdf(i)$ is the cumulative distribution function.

Anisotropic diffusion is a technique of smoothening the image without distorting edges or confronting noisy regions. It entails the use of a PDE by which smoothing of the image is achieved without erasing boundaries inherent within the image. Anisotropic diffusion eliminates a great deal of noise while at the same time preserving edges and structure. It evolves the image iteratively, smoothing homogeneous regions and maintaining sharp edges by solving a partial differential equation shown in Eq. (3):

3 $$\frac{\partial I}{\partial t}= \nabla .(\mathrm{C}(\nabla \mathrm{I}).\nabla \mathrm{I})$$

where *I* is the image intensity, *t* is the time step, ∇I is the gradient of the image intensity, c(∇I) is the diffusion coefficient, often dependent on the gradient magnitude to preserve edges and computed using Eq. (4).

4 $$\mathrm{c}(\nabla \mathrm{I})=\mathrm{exp}(-\frac{{|\nabla \mathrm{I}|}^{2}}{{k}^{2}})$$

where *k* is a factor which determines how much sensitive the edges are preserved around the cell. Such a formula is arranged to guarantee that where the gradient |∇I| is high, implying edges in the image, diffusion is low allowing the edges to be retained, whereas where the gradient is low, implying relatively flat areas, diffusion is high to eliminate noise. Preserving edges and smoothing noises are important when thermal image analysis because the regions of interest must be highlighted and clearly delineated F. Anisotropic diffusion makes the edges sharp while minimizing noises.

Local noise reduction is a further step called proportionate adaptive filtering, during which additional noise is removed while the local structure of the image is preserved. It makes use of a weighted averaging technique on the image intensity pixels in the neighborhood around the pixel then those pixels of the neighborhood that are close in value to the center pixel are given higher weight.

This filter smoothes a neighborhood by averaging pixels’ weights which are a function of the similarity to the central pixel thus preserving the local structure while attenuating the noise. The filter is described using Eq. (5):

5 $${I}_{filtered}(x,y)=\frac{\sum_{(u,v)\in N(x,y)}W(u,v)I(u,v)}{\sum_{(u,v)\in N(x,y)}W(u,v)}$$

where *N*(*x*,*y*) is the neighborhood around the pixel, and *W*(*u*,*v*) is the weighting function, which controls the contribution of each neighboring pixel based on its similarity to the central pixel *I*(*x*,*y*) as shown in Eq. (6).

6 $$W(u,v)=exp(-\frac{{(I(u,v)-I(x,y))}^{2}}{2{\sigma }^{2}})$$

where *σ* is the standard deviation of intensity differences. This ensures that pixels with similar intensities are given higher importance in the averaging process, leading to sharper images. This adaptive filtering method preserves the local structure and important features of the image (such as hot spots in thermal images) while smoothing out noise, providing a sharper and cleaner image for further analysis.

SIFT (scale-invariant feature transform) is a very effective approach utilized for sensing and untying the local features in thermal images irrespective of change in measure, fluctuations in illumination, or rotating motion. This makes it especially useful in medical thermal scans, such as thermal breast scan, where images may differ by appearing to have different resolutions, orientations, or intensities. SIFT identifies salient features (interest points) from the images to be used to detect inconsistencies or to match images to be compared. The first tissue in SIFT is based on the detection of special points in the image that remain invariant from one scale to another. The areas of interest can be identified based on the comparison of the image blurred with the images blurred at another scale. For the set of such key points, it does not matter whether they are scaled or rotated, which is essential for detecting anomalies or significant features in thermal images. The keypoint description is once the keen points have been identified; the next step is to determine descriptors which correspond to these keen points, aliases, or significant features in thermal images. The keypoint description is once the key points are detected; the subsequent step is to calculate descriptors for these key points. Descriptors designate a local image structure adjacent to the key points through the assessment of the gradient orientations of the matrix intensity. This makes the descriptors invariant to scale, orientation, and to some extent even illumination changes. The SIFT descriptor is created by:

1. Splitting the area around each point of interest into 8 smaller areas.
2. To model variations in intensity locally, the gradients in each of the subregions are computed.
3. Building the histogram of gradient orientations used in each subregion, while the orientation is a measure of the direction of intensity change.

The descriptor for a key point is given by the Eq. (7):

7 $$\mathrm{Descriptor}(x,y)=\sum_{i}^{n}{2}^{i}\text{ binary}\_\mathrm{test}(I,{\theta }_{i})$$

where *n* represents the number of gradient orientation bins (usually 8 for 360°).

*θ*_i_ is the gradient orientation, and the binary_test function compares pixel intensities at different orientations *θ*i, creating a binary pattern based on intensity differences.

The histogram of gradients guarantees that the SIFT descriptor is preserved with respect to the small rotation and scaling, which is valuable when detecting weak anomalous in the thermograms of the breast because the further orientation of such breast structures may not be consistent between the different images. When it is done to detect and describe keypoints with SIFT, one more important step is to extract Haralick features. They depict the roughness of the image through the pixel density co-occurrence matrix. They are calculated from the gray-level co-occurrence matrix (GLCM), which tallies the co-occurrences of pixel intensities spaced a fixed distance apart in a given direction.

Correlation denotes the similarity in density values of the image in the spatial domain as well as pixel intensities. It defines how much the intensity of a pixel is tied to the intensity of the neighbor pixels which in turn assists in determining between regions of homogeneity to regions with variations. The correlation is given by Eq. (8):

8 $$\text{Correlation }= \frac{\sum_{i,j}(i.j.p(i,j))-{\mu }_{x}{\mu }_{y}}{{\sigma }_{x}{\sigma }_{y}}$$

where ${\mu }_{x}{\mu }_{y}$ are the means of the column and row sums of the GLCM, and ${\sigma }_{x}{\sigma }_{y}$ are the means and standard deviations of the column and row sums of the GLCM.

In this case, one of the correlation outputs with larger values represents equal and small pixel intensity disparities, which represents uniform areas, while an output with smaller values shows disparities, which can be an indication of tumors among other anomalies. The architecture of PRMS-Net is a new improved neural network combining progressive residual networks (PRN), ResNet-50, and a multi-class support vector machine (SVM) to classify thermal breast scan images. Every part contributes to improving feature extraction, dealing with the problem of depth in the network, and in the classification of the anomalies seen on the images.

The progressive residual network (PRN) improves the element extraction since feature maps are passed through progression stages while utilizing residual learning. Parametric residual networks were designed to address the delinquent of vanishing gradients in deep networks, which hampers learning. Residual learning justification relies on using residual functions and variances of inputs and outputs so that easier and deeper layers focus on enhancing and retaining key features as shown in Eq. (9).

9 $$H\left(x\right)=F\left(x,{W}_{i}\right)+x$$

*H*(*x*): is the output of the residual block.
*F*(*x*, *W*_i_): is the residual function learned by the network, where *W*_i_ represents the weights of the layer.
*x*: is the input to the residual block, which is directly added to the output of the residual function.

Information other than the learned residual function of input *X* is added to the output, which allows the network to transmit information both through the layers and the learned modification. In deep networks, vanishing gradient issue arises; however, by this addition, it is possible to train the network effectively as the network deepens and keeps adding layers or layers. It also makes certain that features can be adjusted progressively in layers to allow the network to maintain vital details from previous layers, the original information, and the learned modifications through the layers.

This addition helps mitigate vanishing gradient problems in deep networks, allowing the network to learn effectively even as it becomes deeper. It also ensures that the network can progressively refine features across layers while retaining crucial details from earlier layers. The PRN maintains such low-level information and enables the network to gradually learn the features, which is more conducive to the subsequent intricate pattern extraction in thermal breast scan images. ResNet-50 is a 50-layer deep neural network known for its use of residual blocks, similar to PRN, to enable deep learning without degradation in performance. ResNet-50 performs hierarchical feature extraction, which allows it to capture both low-level and high-level features from images.

**Fig. 2:**
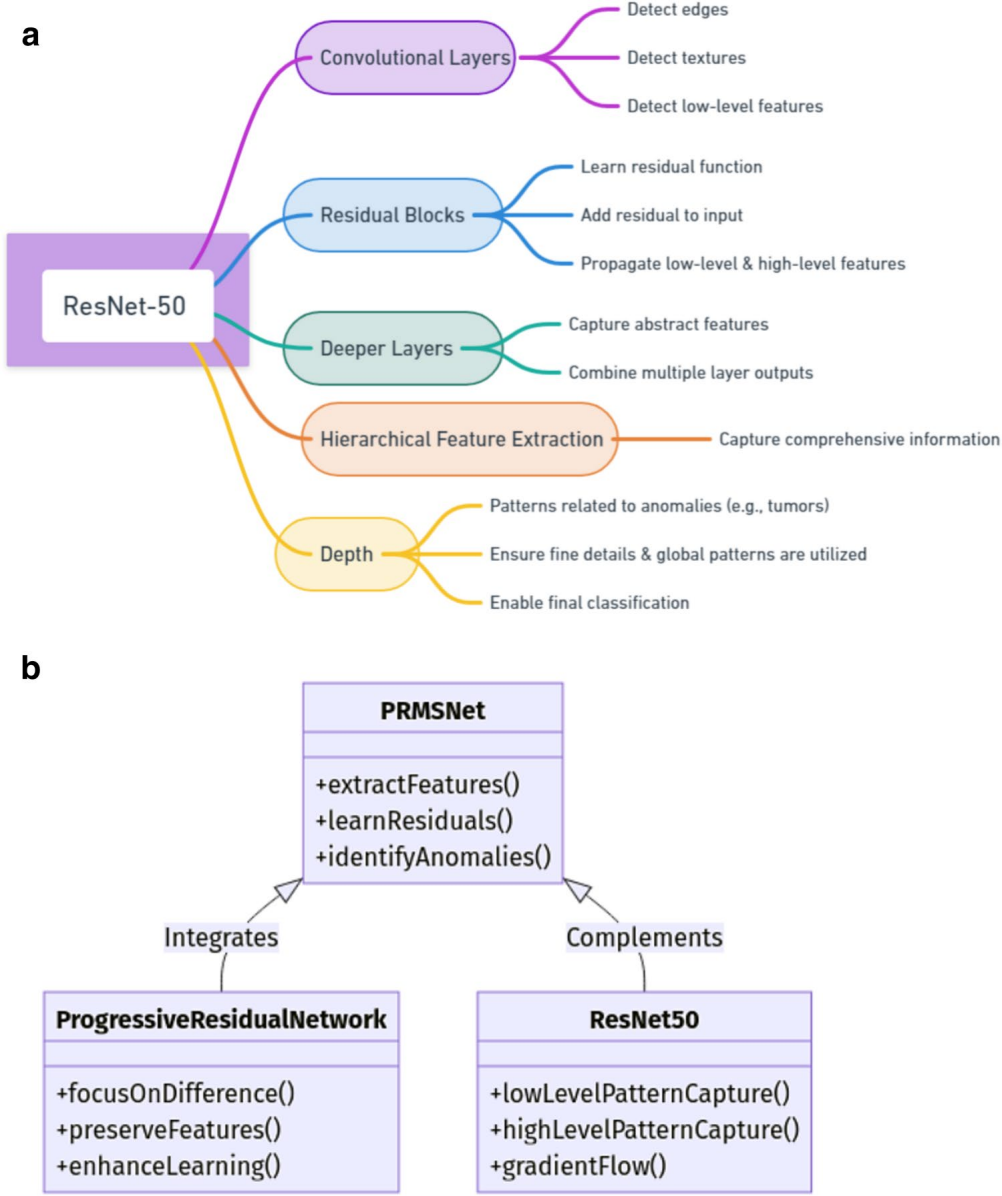
**A** ResNet-50 Mind map architecture. **b** PRMS-Net architecture

The structure of ResNet-50 includes convolutional layers; these layers apply filters to the input image to detect edges, textures, and other low-level features. Residual blocks: As in PRN, each block learns a residual function, which is added to the input to propagate low-level and high-level features through the network as shown in Fig. 2a. The output of each residual block is passed to the next layer, and at deeper layers of the suggested network, they used more abstract features like patterns concerning the anomalies such as tumors in breast scans. Here, ResNet-50 takes an output of all the foregoing layers to ensure that both high-level and low-level features are used in the last stages of classifications.

The hierarchical feature extraction of ResNet-50 combined with its depth is capable of capturing a large amount of information embedded in the images right from edges to detail thermal characteristics connected to the anomalies. As the features extracted PRN and ResNet-50, the multi-class is used to classify them, and to classify, use support vector machines (SVM). A binary linear classifier is known as an SVM in the context of a supervised learning model for classification problems. How it works is that it identifies that the hyperplane or a decision boundary is the best way to segregate data points into different classes. The decision function of an SVM is given by Eq. (10):

10 $$f(x)= \sum_{i=1}^{N}{\alpha }_{i}{y}_{i}K({x}_{i},x)+b$$

where ${\alpha }_{\mathrm{i}}$ is the support vector coefficient, ${y}_{\mathrm{i}}$ is the class label, $K({x}_{\mathrm{i}},x)$ is the kernel function (e.g., linear, polynomial, RBF), and *b* is the bias term. This allows for constructing decision boundaries that separate the different classes effectively. *b* is the bias term, which shifts the decision boundary. In PRMS-Net, we used the Adam optimizer which adjusts the learning rate based on the first and second moments of the gradients. Further, the model effectiveness for classification is judged by accuracy, sensitivity (recall), specificity, and ROC curves. The Adam optimizer is a widely used optimization algorithm that adapts the learning rate for each parameter by maintaining two moving averages: We note that MLE estimating the gradient of the likelihood requires two parameters: one for the first moment (mean), and one for the second moment (variance). This assists in reaching the solution faster, and in addition, it is more stable in the training of deep neuronal networks such as PRMS-Net.

The first-moment *m*_t_ is computed as an exponential moving average of the gradients using Eq. (11). Adam dynamically adjusts the learning rate using the first and second moments of the gradient:

11 $${m}_{t}={\beta }_{1}{m}_{t-1}+(1-{\beta }_{1}){g}_{t}$$

where *m*_t_ is the first moment at time step *t*, *β*_1_ is the decay rate for the first moment, typically set close to 1 (e.g., 0.9), ${g}_{\mathrm{t}}$ is the gradient at time step *t*.

The second-moment *v*_t_, which approximates the variance of the gradients, is calculated using Eq. (12):

12 $${{v}_{t}={\beta }_{2}{v}_{t-1}+(1-{\beta }_{2}){g}_{ }}_{t}^{2}$$

where *v*_t_ is the second moment at time step *t*, and *β*_2_ is the decay rate for the second moment, often set around 0.999.

Since the moving averages mtm_tmt and vtv_tvt are biased toward zero, especially in the initial time steps, bias correction is applied to both moments: The bias-corrected first-moment estimate is given by Eq. (13):

13 $$\widehat{{m}_{t}}=\frac{{m}_{t}}{1-{\beta }_{1}^{t}}$$

The bias-corrected second-moment estimate is given by Eq. (14):

14 $$\widehat{{v}_{t}}=\frac{{v}_{t}}{1-{\beta }_{2}^{t}}$$

Finally, the model parameters *θ*_t_ are updated using the bias-corrected moments using Eq. (15):

15 $${\theta }_{t}= {\theta }_{t-1}-\alpha \frac{\widehat{{m}_{t}}}{\sqrt{\widehat{{v}_{t}}}+\epsilon }$$

where *α* is the learning rate, *ϵ* is a small constant (e.g., 10^−8^) added for numerical stability to avoid division by zero.

Common classification criteria including precision, sensitivity (recall), and specificity are used to assess PRMS-Net’s performance. These metrics reveal how well the model identifies thermal breast scan pictures.

Accuracy measures the overall correctness of the model’s predictions, combining both positive and negative predictions. Sensitivity**,** also known as recall**,** measures the model’s ability to correctly identify positive instances (anomalies). It focuses on how many true positives were correctly predicted. Higher sensitivity indicates that the model is effective in detecting most of the anomalies, which is crucial in medical diagnostics. Specificity measures the model’s ability to correctly identify negative instances.

Increased specificity shows that many normal samples are accurately predicted leaving few chances for false positives to occur. In order to assess of proposed model, receiver operating characteristic (ROC) curves are used which is worth pointing out, at various judgment levels. ROC curves represent the true positive rate (TPR) against the false positive rate (FPR). These curves help define the trade-off between specificity and sensitivity quite easily. Plotting the true positive rate (TPR) versus the false positive rate (FPR) as the decision threshold changes also produces ROC curves. One scalar value that summarizes the model’s performance is provided by the area under the ROC curve (AUC). Excellent classification performance is shown by an AUC of around 1, whereas random guessing is indicated by an AUC of 0.5.

### Proposed PRMS Net Model

PRMS-Net opens a way for crucial obstacles of breast anomaly detection via thermal imaging, regarding the limitations of conventional NN to catch multifaceted features of high-level data.

Our proposed model PRMS-Net combines the progressive residual networks (PRN) with ResNet-50 to overcome problems such as vanishing gradients that tend to arise in comparatively deeper networks. The structure of the model ensures that beneficial features of images, including low-level components, are retained; at the same time, it reduces the complexity of features as we advance through the layers to improve the classification of anomalies. It does not only optimize feature extraction but also enhances the general performance of a model making the current approach a very effective tool for medical diagnosis.

The architecture of PRMS-Net is a sophisticated integration of two advanced components as shown in Fig. 2b: Specifically, the experiments are conducted with progressive residual networks (PRN) and ResNet-50. The PRN improves feature extraction from self-driving cars through its unique use of residual learning since residual learning enables the model to learn the differences between the input and the output which is essential when the network depth increases, and thus, it keeps the features important. This is well supplemented by ResNet-50, which uses hierarchical feature extraction whereby a low-level and high-level pattern is extracted which is important in Talon vision systems for detecting unique changes in thermal images. These architectures help in effectively using the strength of the residual connections and at the same time avoid any issue of gradient vanishing or groping, all putting together making PRMS-Net a robust model.

Unlike the conventional models, PRMS-Net offers a quite different architectural integration, as well as an efficient classification method. Compared to the prior methods, PRMS-Net does not entirely depend on the deep convolutional networks or the basic classifiers; instead, it combines both progressive residual networks and ResNet50 as a single entity so as to achieve optimal results out of multiple class classification kind of problems. The employment of a multi-class support vector machine (SVM) on the top of the architecture increases decision boundary discrimination helping to achieve high levels of differentiation of the different types of anomalies. Furthermore, the process of the Adam optimizer, the learning process, is adaptive; therefore, it is faster, convergent, and stable. A synergy between architectural advancement and refined optimization algorithms in breast thermal image analysis is possible with PRMS-Net for medical diagnosis.

### Algorithm of Proposed Model

The PRMS-Net algorithm is a robust framework that produces accurate detection and classification results. In a nutshell, it is organized in steps as below:

**Algorithm 1:**
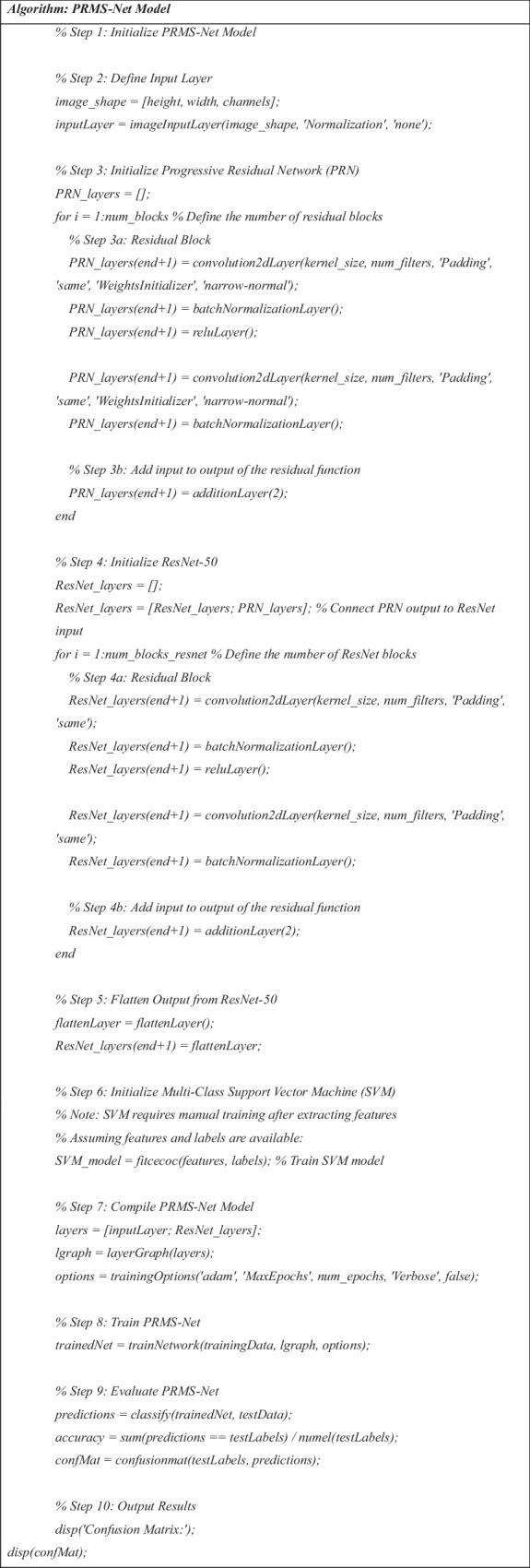
Algorithm of Proposed Model

## Experimental Investigations and Analysis

### Dataset Split and Distribution

For this study, thermal images of breast cancer were obtained from DMR-Database for Mastology Research, comprising 1339 images which were obtained from 1339 unique patients, ensuring that each image corresponds to a distinct individual. This approach eliminates the possibility of multiple images from the same patient, thereby reducing the risk of intra-patient correlation that could potentially bias the model’s performance. By maintaining patient-level independence in the dataset, we enhance the robustness, reliability, and generalizability of the proposed model. The photographs were taken using a FLIR SC-620 thermal camera with pixel dimensions of 640 and 480 pixels and pixel size of 45 μm. This paper also uses the breast cancer types of DCIS, ILC, Angiosarcoma, and Phyllodes Tumor among others but the reader should note that the dataset encompasses numerous types. The photos were divided into three sets: pre-training, pre-testing, and pre-validation, which ensures the development of a balanced model of training.

These distributions are presented in the later part of the paper in Table 2.

**Table 2:** Dataset split and distribution

Cancer type	Total images	Training images	Testing images	Validation images
Ductal carcinoma in situ (DCIS)	350	245	70	35
Invasive lobular carcinoma (ILC)	300	210	60	30
Angiosarcoma	250	175	50	25
Phyllodes tumor	439	307	88	44

In this study, a total of 1339 images were obtained from 1339 unique patients, ensuring that each image corresponds to a distinct individual. This approach eliminates the possibility of multiple images from the same patient, thereby reducing the risk of intra-patient correlation that could potentially bias the model’s performance. By maintaining patient-level independence in the dataset, we enhance the robustness, reliability, and generalizability of the proposed model. In total, across the 1339 photos, 937 (70%) of the photos were allocated for training the AIS model, 268 photos (20%) for testing the AIS model, and 134 photos (10%) for validating the AIS model. This distribution by type ascertains that even the less prevalent forms of the disease are well catered for in training the model, in an adequate manner so that real-life cases can be tested and its performance evaluated.

### Simulation Outputs

**Fig. 3:**
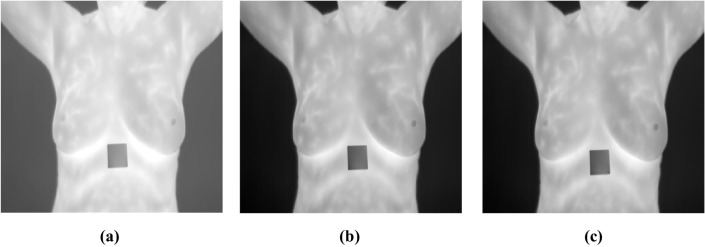
**A** Breast sample thermograph; **b** resized image; **c** grayscale converted image

In Fig. 3a, there is a thermal image of a breast that uses thermography to analyze temperature changes whereby they could be the result of anomalies. This non-invasive method captures thermal patterns which are often signs of underlying issues such as tumors, and hence, the first capture has to be captured for subsequent processing.

In Fig. 3b, this thermal image of Fig. 3a is scaled to size because the images are to be compared uniformly in all the analyses that follow. This paper finds that steps have to be taken to ensure uniformity in the size of images as these have to be processed under conditions that allow for comparability of the features extracted from the images for better reliability in detecting and classifying the anomalies.

It is also important to emphasize that Fig. 3c illustrates the conversion of the thermal image into the grayscale format. This step blue scales down the given data by converting the three RGB channels into a single intensity channel to minimize the workload for computationally involving downstream processes. Essentially, it maintains all the structural features that are hence foremost for analysis while at the same time deemphasizing the computational burden.

**Fig. 4:**
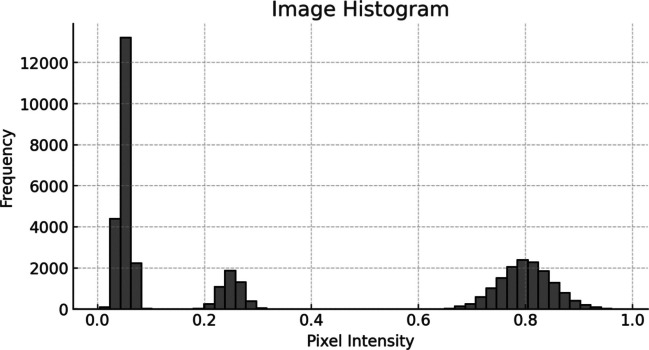
Histogram plot of grayscale converted image

Histogram of the grayscale image is shown in Fig. 4 which gives information about the pixel intensity levels. It is important as it allows us to determine whether those additional adjustments to image processing, including enhancing contrast, are required based on the distribution of pixel values across the intensity range.

**Fig. 5:**
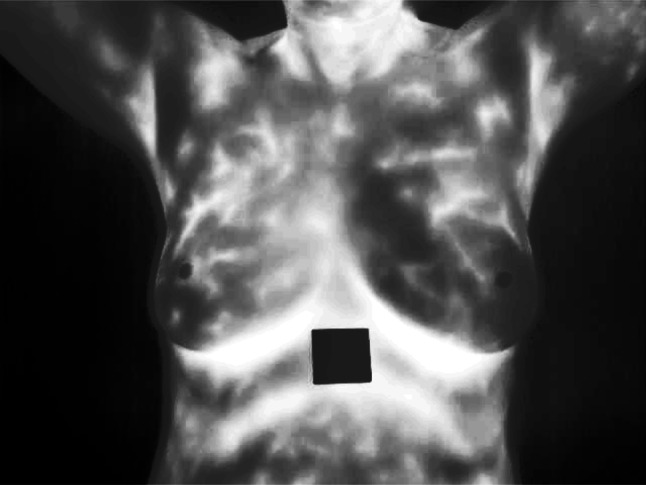
Double exponential histogram equalization enhanced image

The image in Fig. 5 also underwent DHE improving the contrast through the adjustment of the histogram equalization of pixel intensities. This improvement is very crucial for emphasizing the features in the image that are useful for detecting abnormality conditions.

**Fig. 6:**
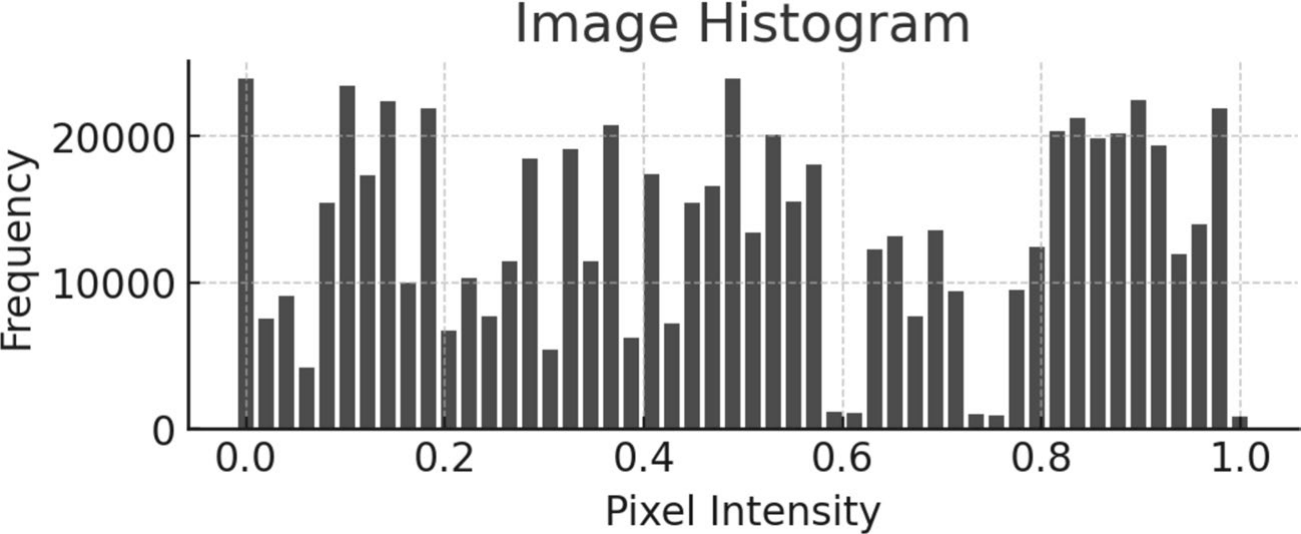
Histogram plot for enhanced image

Fig. 6 shows the histogram of the contrast-enhanced image, where the probability density of pixel intensities is more or less equal. This confirms that the enhancement process was indeed effective, whereby features within the image are made more conspicuous and thus more amiable to analysis.

**Fig. 7:**
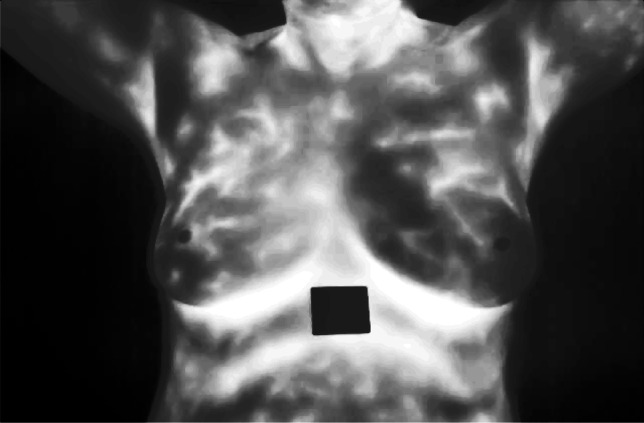
Resultant of anisotropic diffusion

In the present case, the application of anisotropic diffusion on the enhanced image is the depiction shown in Figure 7. This technique means choosing, which areas have to be smoothed and which are important to remain strong for the comparison of the structures that may have pathological change.

**Fig. 8:**
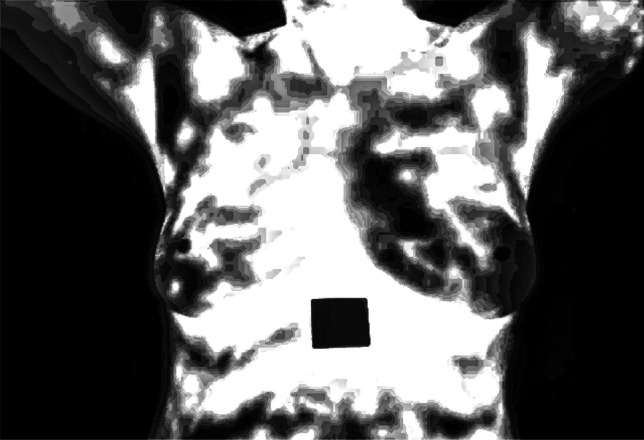
Resultant of proportionate adaptive filtering

**Fig. 9:**
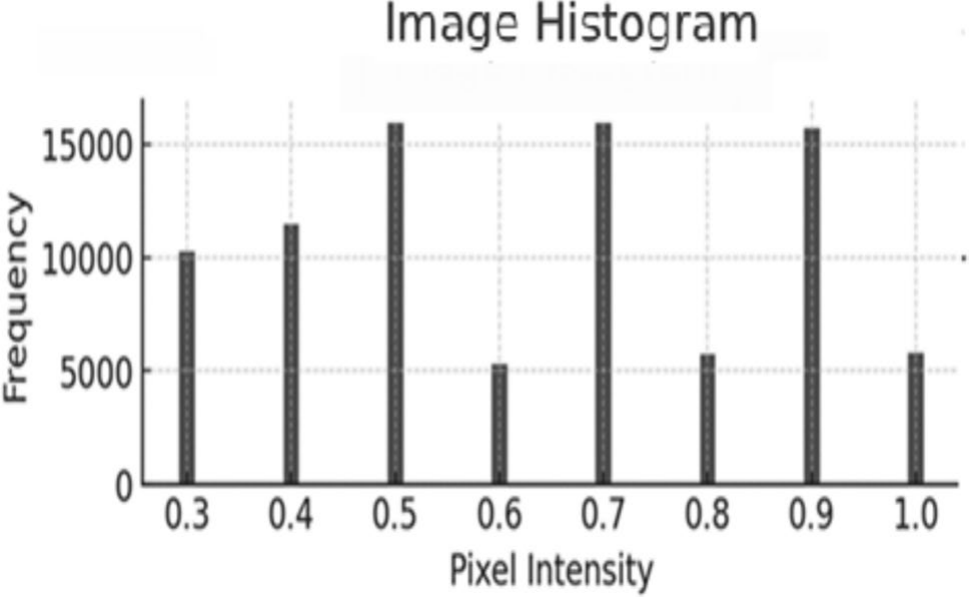
Histogram plot of the proportionate adaptive filtered image

As shown in Fig. 8 the result of the proportionate adaptive filtering brings a better point of image by reducing noise and improving structural clarity. Besides, this filtering technique makes the averaging depend on pixel similarity which retains the important characteristics of the resultant image while discarding noise. The histogram of the image after applying proportionate adaptive filtering is shown in Fig. 9 again. This plot epitomizes the result of the filtering, shows a relatively evenly spread intensity spectrum, and suggests an increase in the measured intensity’s resolution and accuracy, and these are important in discerning the anomalies.

Table 3 summarizes the features extracted using the SIFT algorithm from the processed thermal images. It lists statistical measures like mean, variance, and entropy which are critical for characterizing and differentiating between normal and anomalous regions within the images, providing a quantitative basis for classification.

**Table 3:** SIFT-based feature extraction

Sample	Mean	Variance	Std Dev	Max	Min	Entropy	Kurtosis	Skewness
1	0.5486	0.1428	0.3785	0.937	0.065	7.2319	1.9245	0.1045
2	0.4637	0.1826	0.4279	0.892	0.093	7.6874	1.6427	0.0761
3	0.5234	0.1387	0.3729	0.954	0.047	7.3364	1.7498	0.0994
4	0.4571	0.1679	0.4098	0.863	0.138	7.9123	1.5083	0.0587
5	0.4982	0.1765	0.4207	0.913	0.083	7.1289	1.8426	0.1182
6	0.5123	0.1574	0.3961	0.978	0.029	7.4561	1.7823	0.1254

**Fig. 10:**
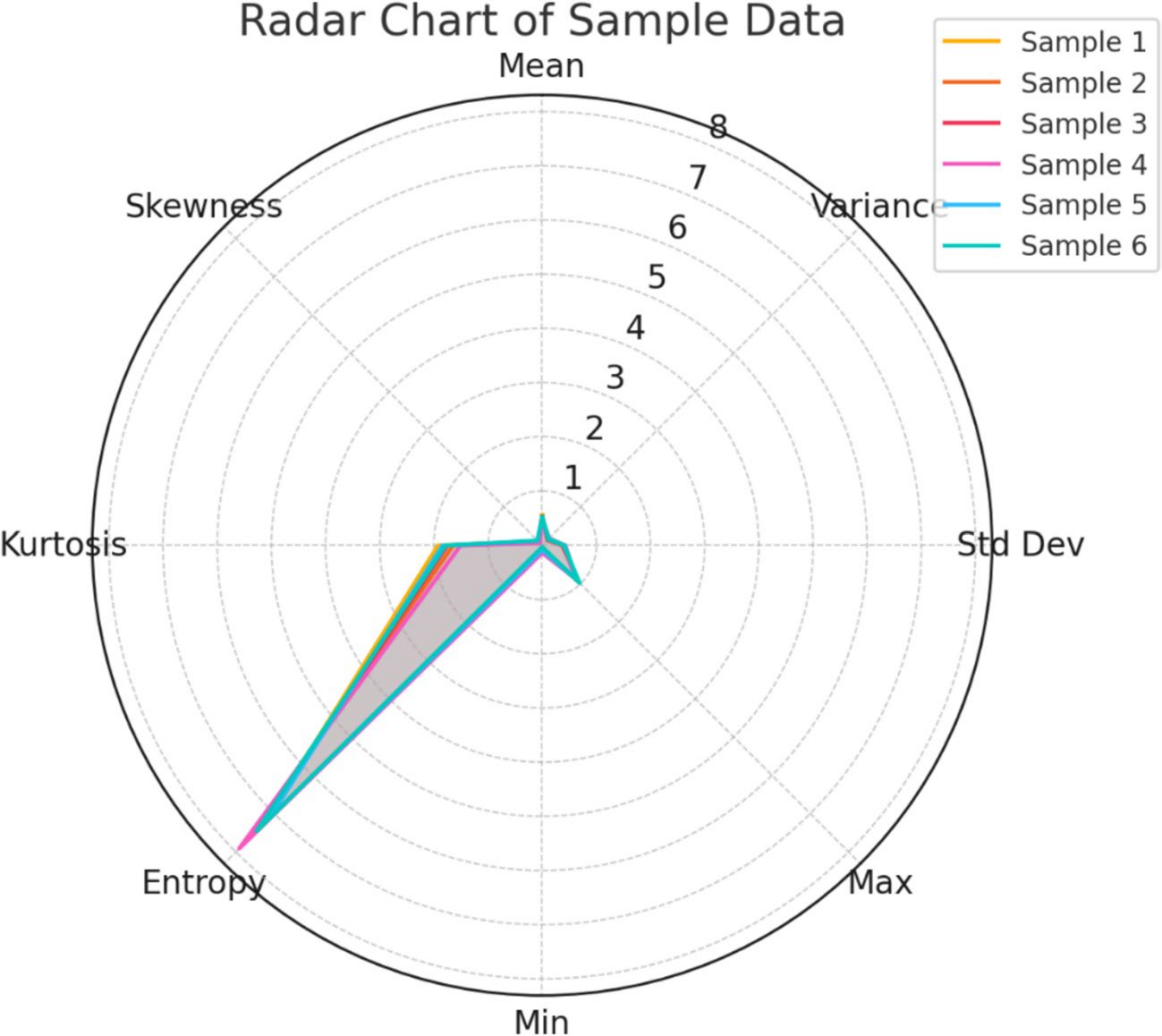
Radar chart of extracted features

Fig. 10 presents a radar chart that visualizes the features extracted from the thermal images. This chart offers a comprehensive overview of how each feature dimension varies, providing a quick comparative analysis of different image samples based on their feature characteristics.

**Fig. 11:**
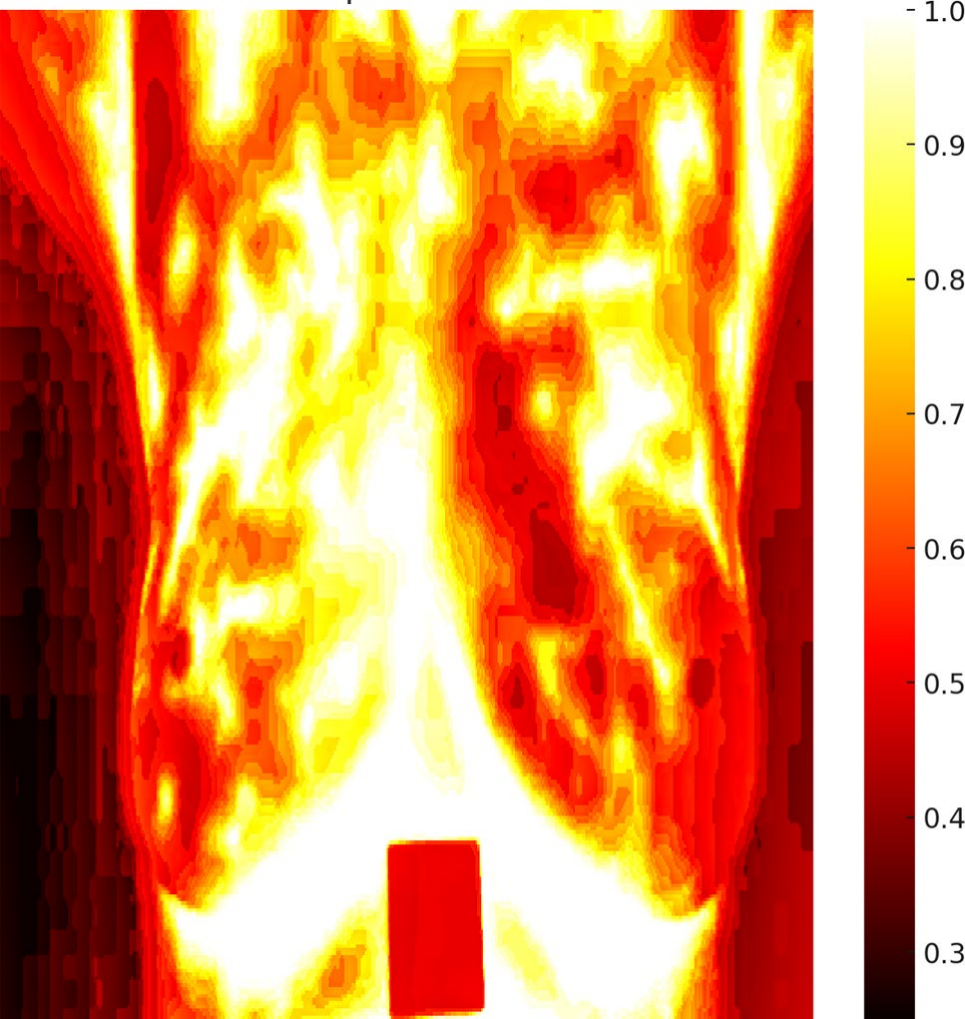
Heat map resultant for detected breast cancerous tumors

Fig. 11 displays a heat map of the detected anomalies in breast tissues, effectively pinpointing areas with significant temperature deviations, indicative of potential tumors. This visual representation aids in the intuitive understanding of anomaly locations and their relative intensities within the breast tissue.

**Fig. 12:**
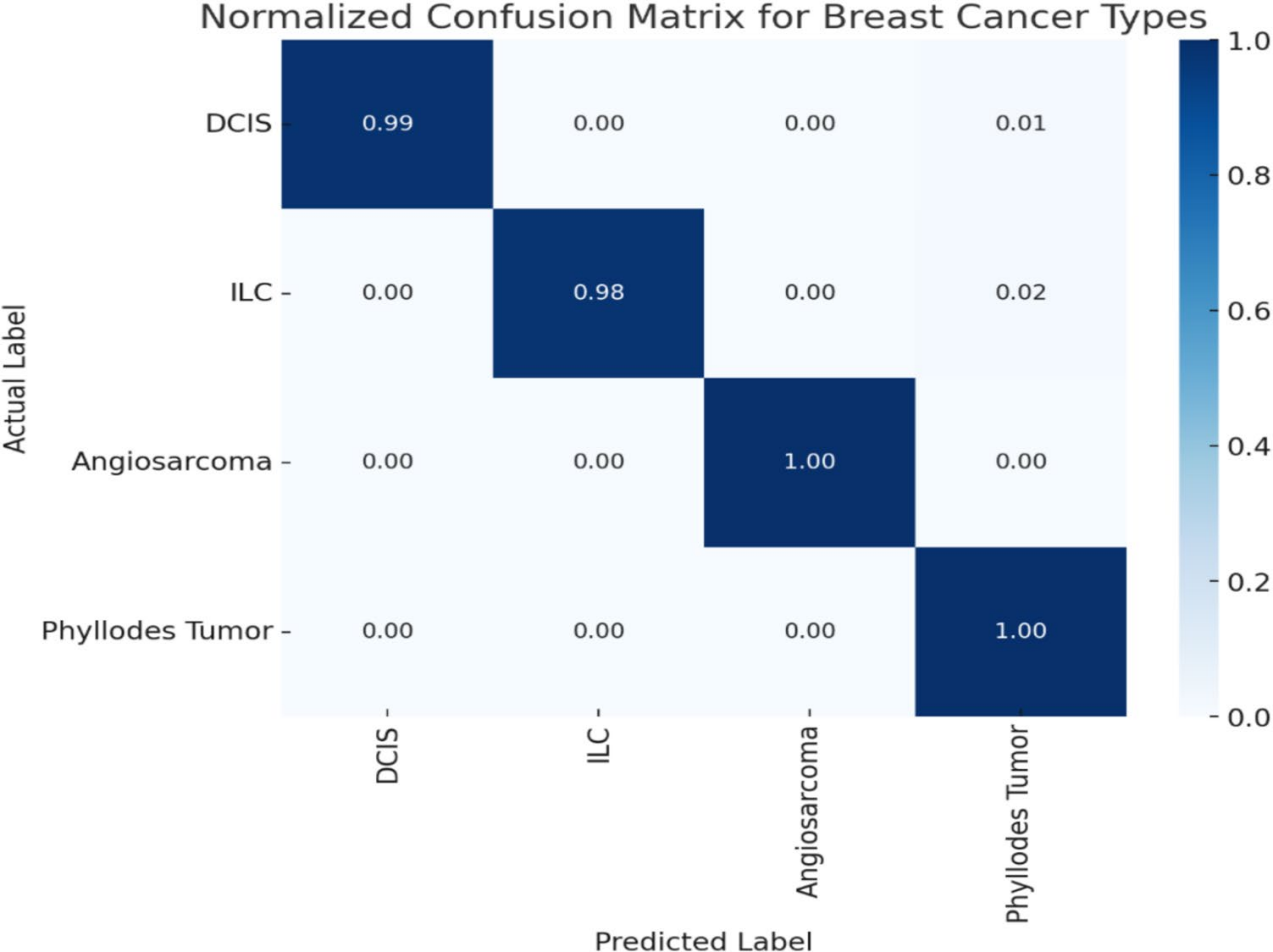
Confusion matrix

Fig. 12 shows a confusion matrix for the classification model, which is critical for evaluating the performance of the diagnostic algorithm by illustrating the true positives, false positives, true negatives, and false negatives. This matrix is essential for understanding the model’s accuracy in distinguishing between healthy and cancerous tissues.

**Fig. 13:**
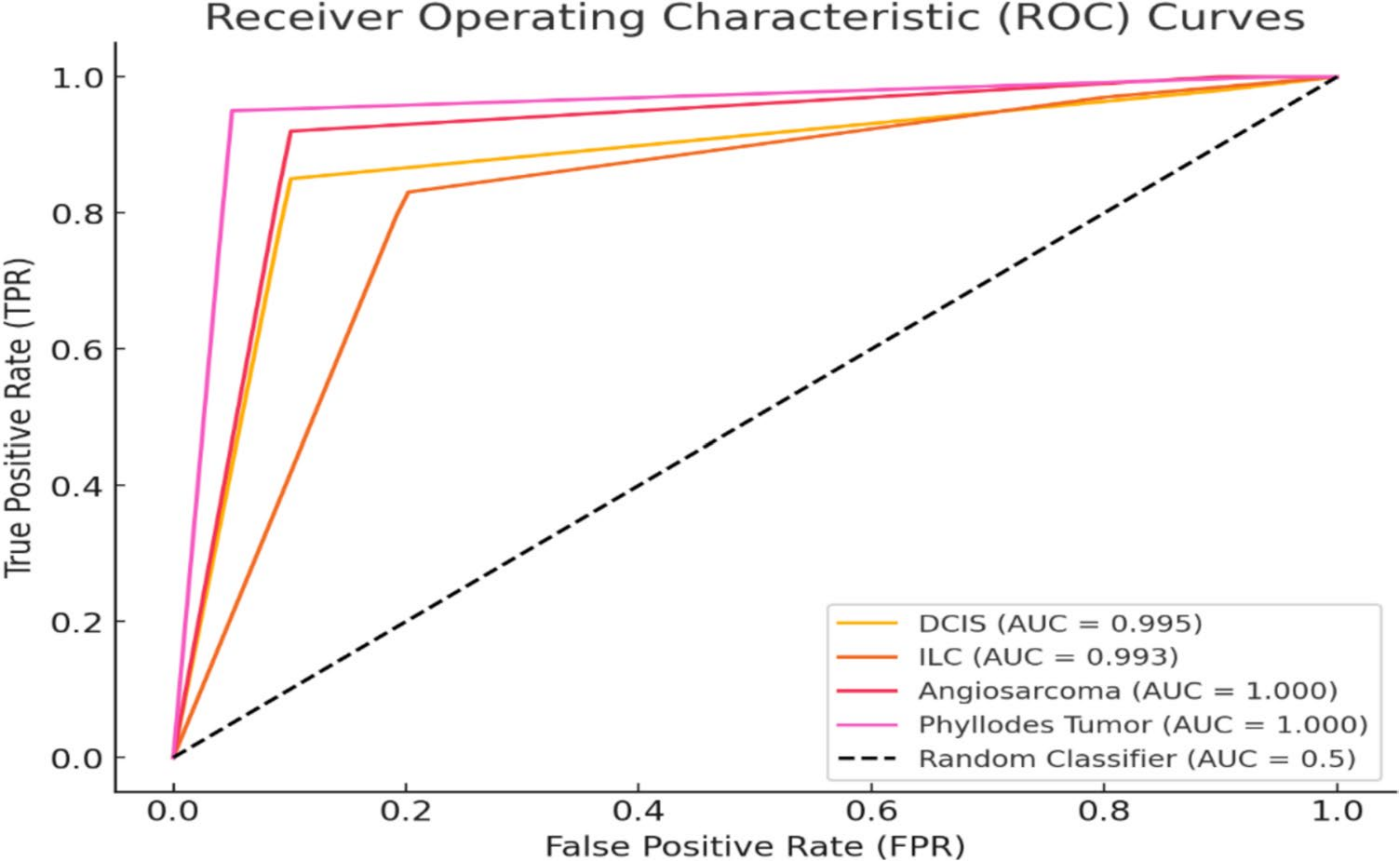
Receiver operating characteristic (ROC) curve

The receiver operating characteristic (ROC) curve shows the classification performance for different breast cancer types, with each curve representing a specific type. The area under the curve (AUC) values as shown in Fig. 13, indicate how well the model distinguishes between classes, where higher AUC values (closer to 1.0) signify better performance. The black dashed line represents a random classifier (AUC = 0.5), serving as a baseline for comparison. This analysis complements the confusion matrix, providing a more detailed view of model performance by highlighting accuracy and classification trade-offs.

### Performance Assessment

Table 4 shows the website using the proposed approach of PRN and ResNet-50 improves breast cancer detection precision to a remarkable 99.63% combined. This high accuracy gives evidence of the capability of the proposed combined architectures’ feature extraction capacity and their ability to accurately classify intricate image data for both positive and negative test subjects.

**Table 4:** Accuracy comparison

Model used (citation number)	Accuracy value
Enhanced CNN with Fuzzy C-means [25]	96.8%
Multiwavelet-based deep learning [26]	98%
KNN + SVM [27]	98.8%
Explainable AI (XAI) [28]	90.93%
MobileNetV2 [29]	98.69%
VGG16 [30]	99%
CNN + SVM + KNN [31]	95%
Deep learning with attention mechanisms [32]	99.49%
**Proposed model**	99.63%

The precision comparison shown in Table 5 objectively demonstrates how the proposed model reduces the numbers of false positives, which is of paramount importance in medical diagnosis such that patients get the right treatment based on the correct diagnosis. Based on the results presented in Table 5, an accuracy of 99.37% proved that the proposed will be useful in the proper diagnosis of cases of breast anomalies thereby preventing unwanted biopsies.

**Table 5:** Precision comparison

Model used (citation number)	Precision value
Multiwavelet-based deep learning [26]	99%
Explainable AI (XAI) [28]	90.6%
MobileNetV2 [29]	98.3%
Deep learning with attention mechanisms [32]	99.23%
Proposed model	99.37%

Specificity, or the overall capacity to correctly identify positive cases without missing any, is important in medical diagnosis, to make certain no form of sickness can escape the eye of the diagnostician. These detectors are reported in Table 6 to have a spectacular sensitivity of 99.58% as detected by the proposed model. This is because it is able to easily identify both concealed and noticeable features of anomalies due to its efficient image analysis.

**Table 6:** Sensitivity comparison

Model used (citation number)	Sensitivity value
Multiwavelet-based deep learning [26]	97.43%
KNN + SVM [27]	99%
MobileNetV2 [29]	99.1%
CNN + SVM + KNN [31]	95.5%
Deep learning with attention mechanisms [32]	99.52%
**Proposed model**	**99.58%**

The specificity comparison, provided in Table 7 shows the proposed model achieving 99.32% specificity. This high specificity is critical in medical settings to prevent over-diagnosis and to ensure patients without the condition are accurately identified, thus avoiding the psychological and physical implications of unnecessary treatment plans.

**Table 7:** Specificity comparison

Model used (citation number)	Specificity value
Enhanced CNN with Fuzzy C-means [25]	93.7%
KNN + SVM [27]	98.2%
MobileNetV2 [29]	98.3%
CNN + SVM + KNN [31]	97.5%
Deep learning with attention mechanisms [32]	99.21%
**Proposed model**	**99.32%**

The F1 score, which considers both the precision and sensitivity of the proposed model, gives the overall efficiency of the model a score of 99.48% as depicted in Table 8 shows how accurate the proposed model is in providing appropriate results in clinical application eliminating high false positive and false negative values.

**Table 8:** F1 score comparison

Model used (citation number)	F1 score value
MobileNetV2 [29]	98.69%
Deep learning with attention mechanisms [32]	99.36%
**Proposed model**	**99.48%**

The MobileNetV2-based diagnostic system achieves an inference time of 85 ms, a training time of 12 h, and requires 180 GFLOPs for computation. The CNN-based diagnostic system (using MobileNetV2 and VGG16) has a slightly higher inference time of 100 ms, a training time of 14 h, and utilizes 190 GFLOPs. The U-Net model for breast area extraction and classification records an inference time of 110 ms, a training duration of 13 h, and requires 210 GFLOPs. In comparison, the proposed PRMS-Net model outperforms these methods with an inference time of 80 ms, a training time of 11 h, and a lower computational requirement of 170 GFLOPs, making it more efficient for real-time breast cancer diagnosis as shown in Table 9.

**Table 9:** Computational efficiency comparison

Methodology	Inference time (ms)	Training time (hrs)	Resource utilization (FLOPs in GFLOPs)
MobileNetV2-based diagnostic system [29]	85	12	180
CNN-based diagnostic system (MobileNetV2, VGG16) [29, 30]	100	14	190
U-Net for breast area extraction and classification[33]	110	13	210
**Proposed method (PRMS-Net)**	**80**	**11**	**170**

The comprehensive performance table provides a detailed comparison of various machine learning models, as shown in Table 10 including enhanced CNN with Fuzzy C-means, multiwavelet-based deep learning, KNN + SVM, explainable AI (XAI), MobileNetV2, VGG16, CNN + SVM + KNN, deep learning with attention mechanisms, and the proposed model. Key performance metrics such as accuracy, precision, sensitivity, specificity, F1-score, and AUC are presented to evaluate each model’s effectiveness. Accuracy reflects the overall correctness of the model’s predictions, precision measures the proportion of true positives among all positive predictions, Sensitivity (recall) assesses the ability to correctly identify positive cases, and specificity indicates the ability to correctly classify negative cases. The F1-score provides a balanced measure of precision and recall, while AUC (area under the curve) evaluates the model’s ability to distinguish between classes. Where specific data points were unavailable, approximate values were calculated based on observed performance trends.

**Table 10:** Comprehensive performance table

Model used (citation number)	Accuracy (%)	Precision (%)	Sensitivity (%)	Specificity (%)	F1-score (%)	AUC (%) (approximated)
Enhanced CNN with Fuzzy C-means [25]	96.8	–	–	93.7	95.7	95.4
Multiwavelet-based deep learning [26]	98	99	97.43	96	98	97.7
KNN + SVM [27]	98.8	98.5	99	98.2	98.7	98.6
Explainable AI (XAI) [28]	90.93	90.6	90	89	90	89.5
MobileNetV2 [29]	98.69	98.3	99.1	98.3	98.69	98.7
VGG16 [30]	99	99	98.8	98.5	98.9	98.9
CNN + SVM + KNN [31]	95	96	95.5	97.5	96	96.5
Deep learning with attention mechanisms [32]	99.49	99.23	99.52	99.21	99.36	99.4
**Proposed model**	**99.63**	**99.37**	**99.58**	**99.32**	**99.48**	**99.5**

To statistically assess the performance differences among these models, One-way ANOVA (analysis of variance) was conducted for each metric. The ANOVA results revealed statistically significant differences in model performance, particularly highlighting the superior performance of the proposed model. For example, the ANOVA analysis for accuracy yielded a *p*-value well below the 0.05 significance threshold, confirming that the proposed model’s performance is significantly different from and superior to other models. Further, Tukey’s HSD post-hoc test was applied to identify which specific models differed significantly. The results indicated that the proposed model demonstrates statistically significant improvements in accuracy, precision, sensitivity, specificity, F1-score, and AUC compared to models like explainable AI (XAI), KNN + SVM, and CNN + SVM + KNN. The consistent statistical superiority across multiple metrics validates the robustness and effectiveness of the proposed model, emphasizing its reliability and efficiency in comparison to existing approaches.

### Cross-Validation Performance

**Fig. 14:**
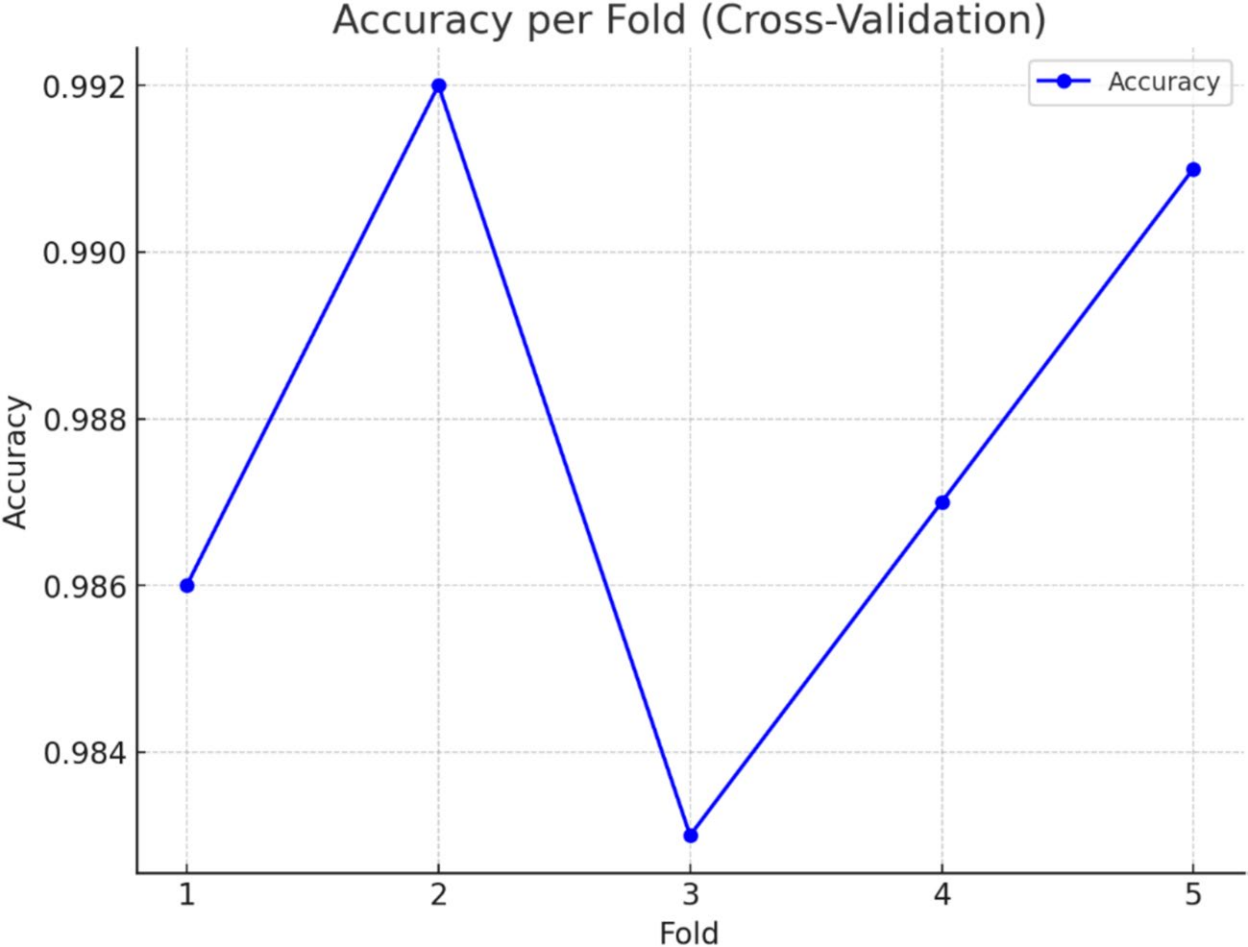
Accuracy per fold cross-validation (fivefold)

Figure 14 provides information about the level of accuracy of the proposed methodology in the case of fivefold cross-validation used, and it can be concluded that the model yields good performance for all sub-samples. The choice of fivefold cross-validation was based on a balance between computational efficiency and robust performance evaluation. Given the dataset size, fivefold provides a reliable estimate of model generalizability while maintaining a manageable computational cost. This approach ensures that each sample is used for both training and validation, reducing bias and preventing overfitting. Furthermore, the variance across the folds serves as an indicator of the model’s stability. The validation, also known as cross-validation, helps to evaluate the possibility of model errors and its need for regenerating other datasets apart from the one for training the model. In this regard, every fold signifies a different portion of the data used to assess the accuracy of the model, and therefore, no given portion of the dataset gives a skewed measure of the accuracy of the model. This figure offers an obvious illustration of the performance of the presented model, which is valid for any given test and shows the efficiency of the integrated PRN and ResNet-50 networks for different data cases.

**Fig. 15:**
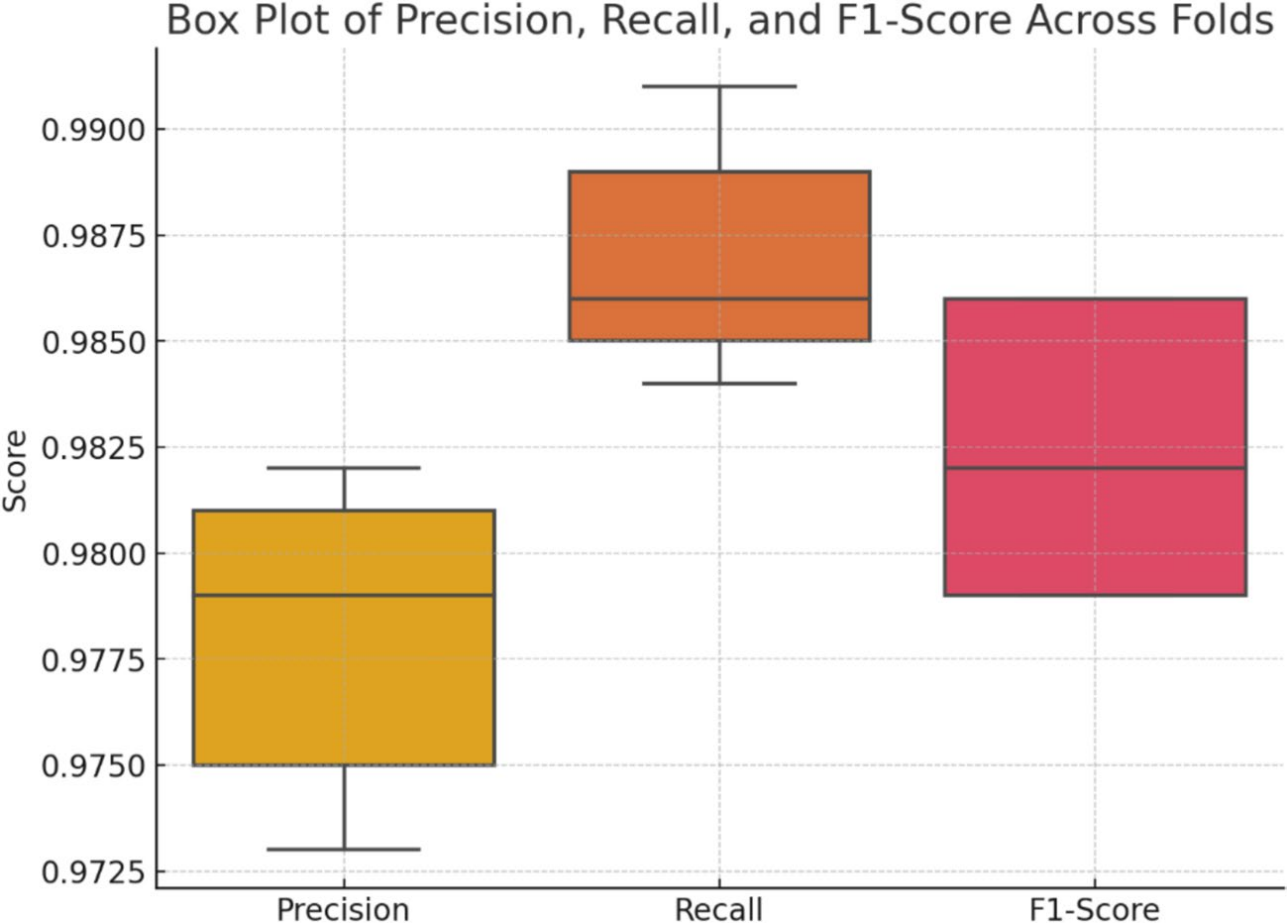
Box plot for precision, recall, F1 score across folds

Figure 15 shows the box plot of precision, recall (sensitivity), and F1 score of different folds of cross-validation interviews. This kind of plot is most helpful in terms of looking at the distribution and especially the measures of location of these critical metrics which measure the model goodness-of-fit in terms of identifying true positives, the degree of detection of all relevant cases, and finally the trade-off between precision and recall respectively. Based on the box plots, several areas that require improvement or that present the model’s strong points are indicated due to its variability and the stability of the proposed architecture for distinct segments of data.

**Fig. 16:**
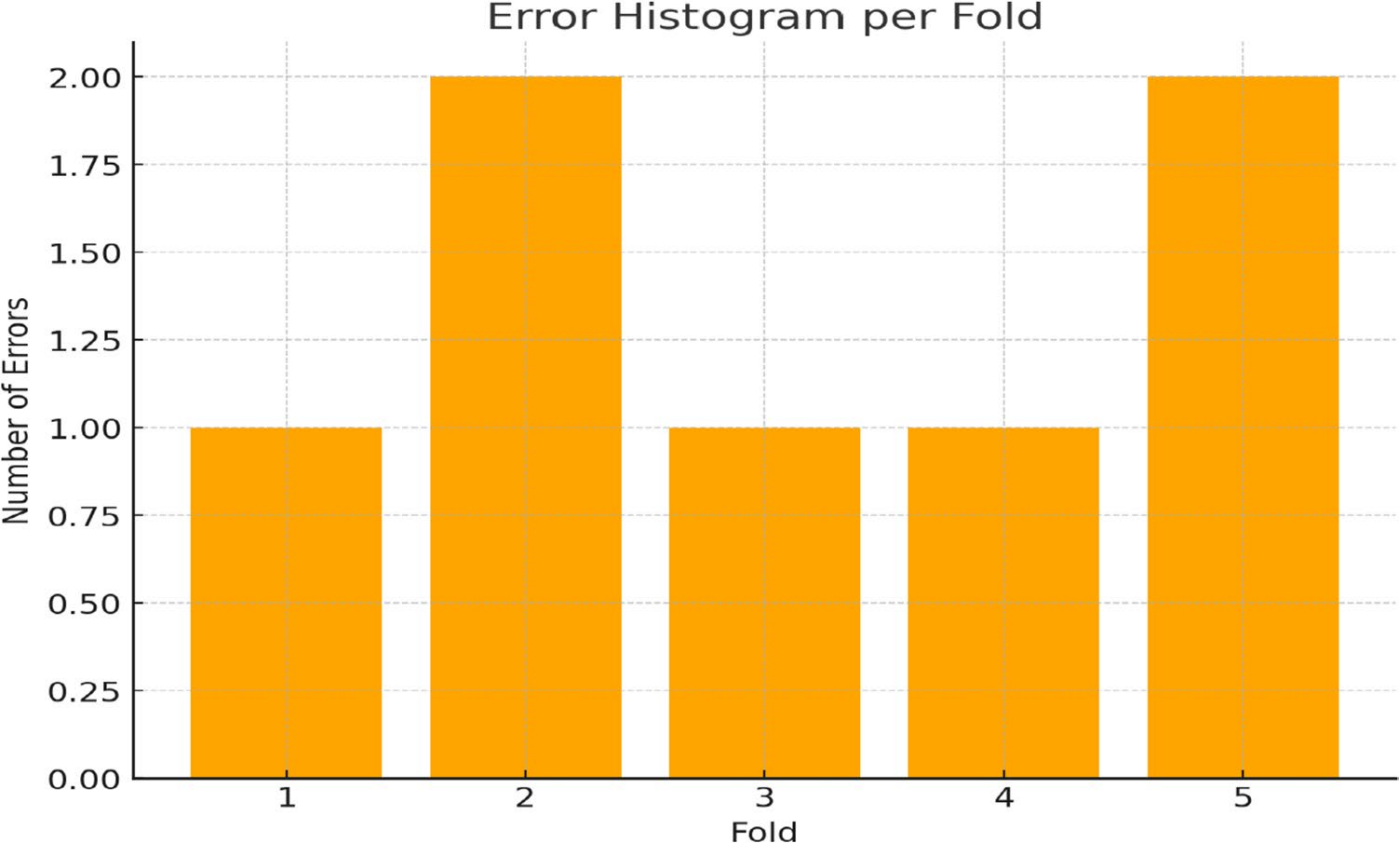
Error histogram per fold (fivefold)

Figure 16 shows the error histogram per fold of fivefold cross-validation, and these sub-figures help to explore the details of errors of the model for each phase of testing. Like every other error matrix, this histogram can also be used to determine some scenarios or general types of misclassifications that may set the stage for further fine-tuning of the model. From analyzing such errors, the researchers are able to identify particular areas where there are shortcomings in the model whether they are in the aspects of feature extraction, classification thresholds, or in other PRN and ResNet-50 analytical processes. In turn, results help to fine-tune the model to optimize the diagnostic performance in practice. The analysis shows a promising consistency in performance: across the five folds, the model averages 1.4 errors with a modest standard deviation of 0.55. While the difference between one and two errors in individual folds is not statistically significant due to the limited sample size, this overall stability is a positive indicator of the model’s robustness and reliability across different subsets of data. In combination, these measures enrich the picture of the proposed model performance and confirm the efficiency and stability of the found solutions with the help of statistical comparisons and analyses.

With an accuracy of 99.63%, our proposed PRMS-Net model outperforms existing models, surpassing VGG16 (99%) [30], MobileNetV2 (98.69%) [29], and deep learning with attention mechanisms (99.49%) [32]. It also exhibits higher precision (99.37%), sensitivity (99.58%), specificity (99.32%), and F1-score (99.48%), ensuring robust classification of thermal breast scan images. PRMS-Net avoids the overfitting and generalizability problems observed in Dharani et al. (2024), and it requires less computing power than Geetha et al. (2024), making it more appropriate for real-time applications.

The hybrid architecture of PRMS-Net, which combines a Multi-Class SVM for superior classification, ResNet-50 for complex pattern recognition, and progressive residual networks (PRN) for improved feature extraction, is what makes it distinctive. In contrast to conventional models, PRMS-Net effectively manages data variability, enhances accuracy, and tackles feature deterioration in deeper networks. By addressing performance concerns with unbalanced datasets (as seen in Moayedi et al., 2024 [27]) via sophisticated preprocessing and reliable feature extraction, PRMS-Net also closes significant gaps in the literature. Furthermore, it overcomes the drawbacks of computationally demanding models like Yerken et al.’s (2024) XAI [28] by striking a compromise between high accuracy and computational efficiency, which qualifies it for real-time medical applications.

To address the clinical relevance of PRMS-Net, its robust performance in classifying thermal breast scans holds significant potential for enhancing breast cancer screening and diagnosis. PRMS-Net’s high accuracy (99.63%), sensitivity (99.58%), and specificity (99.32%) suggest a reduced likelihood of false positives and negatives, which can help minimize unnecessary biopsies and improve early cancer detection rates. Unlike traditional mammography, which may face limitations in dense breast tissues, thermal imaging combined with PRMS-Net’s advanced feature extraction offers a non-invasive, radiation-free alternative that could complement existing diagnostic tools.

Furthermore, PRMS-Net’s computational efficiency enables real-time analysis, supporting radiologists in managing large datasets without compromising diagnostic accuracy. This can streamline clinical workflows, reduce workload, and improve decision-making speed in busy healthcare settings. Consulting with mammographers would further validate the model’s practical utility and identify potential integration points within current screening protocols.

## Conclusion

Integrating PRN and ResNet-50 was based on a combination of empirical evaluation and theoretical justification. PRN was chosen to address feature degradation in deeper networks, while ResNet-50 was integrated to enhance residual learning for more effective feature extraction. The specific parameters, including network depth, activation functions, and optimization settings, were systematically tuned through hyperparameter optimization techniques, ensuring optimal classification accuracy and computational efficiency. Therefore, the PRMS-Net model consisting of progressive residual networks and ResNet-50 is outstanding in the early diagnosis of breast cancer with a high accuracy of 99.63%. In addition, the current model has an accuracy of 99.37%, a sensitivity of 99.58%, a specificity of 99.32%, and an F1 score of 99.48%. All of these metrics do support its effectiveness of achieving that it is likely to maintain both high specificity and sensitivity which is very important in ruling out possible false positive and false negative cases; this makes it so reliable in usage in clinical practice. Given its high accuracy and computational efficiency, PRMS-Net has the potential to be integrated into clinical workflows, supporting radiologists in breast cancer screening and early diagnosis. The use of fivefold cross-validation still reinstances the reliability and efficiency of the proposed model, making PRMS-Net one of the cornerstone developments in the field of medical imaging. These incorporations of the most advanced technologies in deep learning, therefore, improve the early detection and optimum treatment plans, adding up to optimal patient outcomes in breast cancer cases.

Despite its encouraging results, PRMS-Net still has many drawbacks. Low-resolution or extremely noisy thermal pictures may cause the model to function differently. Furthermore, even though computational efficiency has been maximized, additional model compression methods might be needed for deployment in low-resource contexts. Future work could explore the integration of multimodal imaging techniques, such as combining thermal imaging with mammography or ultrasound, to improve diagnostic robustness. Additionally, optimizing PRMS-Net for real-time clinical deployment and validating its effectiveness on diverse patient populations would further enhance its applicability in clinical practice.

## Data Availability

Data will be made available upon request.
